# Cholesterol and 27-hydroxycholesterol promote thyroid carcinoma aggressiveness

**DOI:** 10.1038/s41598-019-46727-2

**Published:** 2019-07-16

**Authors:** Giovanna Revilla, Monica de Pablo Pons, Lucía Baila-Rueda, Annabel García-León, David Santos, Ana Cenarro, Marcelo Magalhaes, R. M. Blanco, Antonio Moral, José Ignacio Pérez, Gerard Sabé, Cintia González, Victoria Fuste, Enrique Lerma, Manuel dos Santos Faria, Alberto de Leiva, Rosa Corcoy, Joan Carles Escolà-Gil, Eugenia Mato

**Affiliations:** 1 Department of Endocrinology-EDUAB-HSP, Hospital de la Santa Creu i Sant Pau, Barcelona, Spain; 2 Institut d’Investigacions Biomèdiques (IIB) Sant Pau, Hospital de la Santa Creu i Sant Pau, Barcelona, Spain; 3 Departament de Bioquímica, Biologia Molecular i Biomedicina, Universitat Autònoma de Barcelona, Barcelona, Spain; 4 Unidad Clínica y de Investigación en Lípidos y Arteriosclerosis, Hospital Universitario Miguel Servet, Instituto de Investigación Sanitaria Aragón (IIS Aragón), Zaragoza, Spain; 5 CIBER de Enfermedades Cardiovasculares, CIBERCV, Madrid, Spain; 6 CIBER de Diabetes y Enfermedades Metabólicas Asociadas, CIBERDEM, Madrid, Spain; 7 CIBER Bioingeniería, Biomateriales y Nanomedicina, CIBER-BBN, Madrid, Spain; 8 Service of Endocrinology, Clinical Research Center (CEPEC), Hospital of the Federal University of Maranhão (HUUFMA), São Luís, Maranhão, Brazil; 9 Department of General Surgery—Hospital de la Santa Creu i Sant Pau, Barcelona, Spain; 10 Medicine Department, Autonomous University of Barcelona (UAB), Barcelona, Spain; 11 Department of Anatomic Pathology—Hospital de la Santa Creu i Sant Pau, UAB, Barcelona, Spain

**Keywords:** Lipoproteins, Thyroid cancer

## Abstract

Cholesterol mediates its proliferative and metastatic effects via the metabolite 27-hydroxycholesterol (27-HC), at least in breast and endometrial cancer. We determined the serum lipoprotein profile, intratumoral cholesterol and 27-HC levels in a cohort of patients with well-differentiated papillary thyroid carcinoma (PTC; low/intermediate and high risk), advanced thyroid cancers (poorly differentiated, PDTC and anaplastic thyroid carcinoma, ATC) and benign thyroid tumors, as well as the expression of genes involved in cholesterol metabolism. We investigated the gene expression profile, cellular proliferation, and migration in Nthy-ori 3.1 and CAL-62 cell lines loaded with human low-density lipoprotein (LDL). Patients with more aggressive tumors (high-risk PTC and PDTC/ATC) showed a decrease in blood LDL cholesterol and apolipoprotein B. These changes were associated with an increase in the expression of the thyroid’s LDL receptor, whereas 3-hydroxy-3-methylglutaryl-CoA reductase and 25-hydroxycholesterol 7-alpha-hydroxylase were downregulated, with an intratumoral increase of the 27-HC metabolite. Furthermore, LDL promoted proliferation in both the Nthy-ori 3.1 and CAL-62 thyroid cellular models, but only in ATC cells was its cellular migration increased significantly. We conclude that cholesterol and intratumoral accumulation of 27-HC promote the aggressive behavior process of PTC. Targeting cholesterol metabolism could be a new therapeutic strategy in thyroid tumors with poor prognosis.

## Introduction

Cancer is a multifactorial disease that is associated with important metabolic disorders, one of which is the metabolism of cholesterol. Cholesterol molecules and their metabolites play important roles in normal cells, actively participating in the formation of cell membranes, as well as the cell cycle, and the tumoral cells require an increased supply of cholesterol. To meet these needs, these deregulated cells are able to promote two strategies cholesterol uptake from the bloodstream and *de novo* cholesterol biosynthesis^1–4^. In this context, hypercholesterolemia has been identified as a risk factor in the development or malignant drift of some solid tumors, such as in breast, colon, and prostate cancer^5–8^. In contrast, the inhibition of the cholesterol synthesis pathway can activate the mechanisms of cell differentiation^9^.

One feature of some malignant tumors is the existence of an imbalance between the accumulation of intracellular cholesterol and inhibition of the cholesterol storage machinery^10–12^. Other processes related to the malignant drift of the tumors, such as cell migration and metastasis, have been demonstrated to be dependent on intracellular accumulation of cholesterol^3,6,7^. Moreover, oxysterols are oxidized derivatives of cholesterol, and they are involved in the regulation of the cholesterol metabolism; oxysterols are associated with different chronic diseases, such as atherosclerosis, neurodegenerative diseases, and inflammatory bowel diseases^13^. Furthermore, the oxysterols have also been related to the development of cancer trough the hedgehog, WNT, and MAPK signaling pathways^14,15^. Different forms of oxysterols have been identified; the most important are 27-, 24-, and 7*α*-hydroxycholesterol. 27-hydroxycholesterol (27-HC), which is mainly synthesized in the liver, acts as a potent suppressor of cholesterol synthesis throughout the sterol regulatory element-binding proteins (SREBPs)^16^. 27-HC levels are controlled by two mitochondrial enzymes, namely sterol 27-hydroxylase (*CYP27A1)*—which is responsible for their synthesis—and 25-hydroxycholesterol 7-alpha-hydroxylase (*CYP7B1*), which is responsible for their catabolism^17^. Different studies have demonstrated the link among the levels of 27-HC, tumor proliferation and estrogen receptor expression in breast cancer^15,18–20^. The analysis of both genes in breast tumors with positive estrogen receptors showed that the prognosis and survival of patients were independent of the *CYP27A1* gene expression, while low expression levels of the *CYP7B1* gene were correlated with worse patient survival^15,19,21^. 27-HC can activate the liver X receptors (LXRs), which may regulate the target genes controlling cholesterol, glucose and fatty acid metabolism, as well as inflammatory responses and the expression of specific genes that control cell proliferation and metastasis processes^22,23^.

Several epidemiological studies have investigated the link between dyslipidemias, high-fat diets, and cancer^24–26^. In thyroid cancer (TC), this relationship is still unknown, although some evidence in the last decade suggests that the body fat percentage, cholesterol levels, and adiposity could be associated with the increased incidence of TC, papillary subtype (PTC)^27,28^. However, the effects of the cholesterol metabolism and metabolite 27-HC on TC progression remain unknown. The objective of this study was to analyze the associations between cholesterol, intratumoral 27-HC levels, and TC tumors’ malignancy.

## Results

### Low LDL-cholesterol levels in patients with high-risk PTC and dedifferentiated tumors

The serum lipid profiles of patients with different thyroid neoplasms are summarized in Table 1. Patients with high-risk PTC and PDTC/ATC tumors showed lower serum cholesterol levels (3.832 ± 0.198 mmo/L, *p* = 0.0041 and 3.793 ± 0.245 mmo/L, *p* = 0.0293, respectively) compared with those with BTT tumors (4.933 ± 0.207 mmo/L) and low/intermediate-risk PTC (4.581 ± 0.159 mmo/L). These changes were associated with a decrease in LDL cholesterol in high-risk PTC tumor patients (1.944 ± 0.222 mmo/L, *p* = 0.0515) and those with dedifferentiated PDTC/ATC tumors (1.775 ± 0.083 mmo/L, *p* = 0.0274) compared with those with BTT (2.551 ± 0.165 mmo/L) and low/intermediate-risk patients PTC (2.293 ± 0.129 mmo/L). The apoB levels were also decreased in patients with high-risk PTC tumors (0.748 ± 0.07 mmo/L, *p* = 0.0068) and PDTC/ATC (0.714 ± 0.064 mmo/L, *p* = 0.011). However, the HDL cholesterol levels did not differ among the groups analyzed. Moreover, the triglyceride levels were reduced in patients with all the types of malignant tumors, but significant changes were only found in the high-risk PTC group (0.824 ± 0.101 mmo/L, *p* = 0.0015).

**Table 1:** Patients’ serum lipid profile according to histology pattern.

	BTT	Low/intermediate-risk PTC	High-risk PTC	PDTC/ATC
Cholesterol (mmol/L)	4.933 ± 0.207	4.581 ± 0.159	3.832 ± 0.198*	3.793 ± 0.245*
Triglycerides (mmol/L)	1.563 ± 0.156	1.290 ± 0.105	0.824 ± 0.101**	0.936 ± 0.164
Phospholipids (mmol/L)	2.824 ± 0.101	2.558 ± 0.088	2.182 ± 0.112**	2.321 ± 0.131*
HDL Cholesterol (mmol/L)	1.673 ± 0.132	1.704 ± 0.106	1.515 ± 0.143	1.593 ± 0.267
LDL Cholesterol (mmol/L)	2.551 ± 0.165	2.293 ± 0.129	1.944 ± 0.222	1.775 ± 0.083*
ApoB (g/L)	0.990 ± 0.044	0.961 ± 0.037	0.748 ± 0.07**	0.714 ± 0.064*

Mean ± SEM of total cholesterol, triglycerides, phospholipids, low-density lipoprotein cholesterol, high-density lipoprotein cholesterol, apolipoprotein (apo) B in BTT (*n* = 27), low/intermediate-risk PTC (*n* = 43), high-risk PTC (*n* = 12), and PDTC/ATC (*n* = 7). Analysis of variance using ANOVA plus Tukey’s post-hoc test (*p < 0.05, **p < 0.01). P-values are in comparison to BTT.

### The LDL receptor (LDLR) is upregulated in tumors with higher malignancy

**Figure 1:**
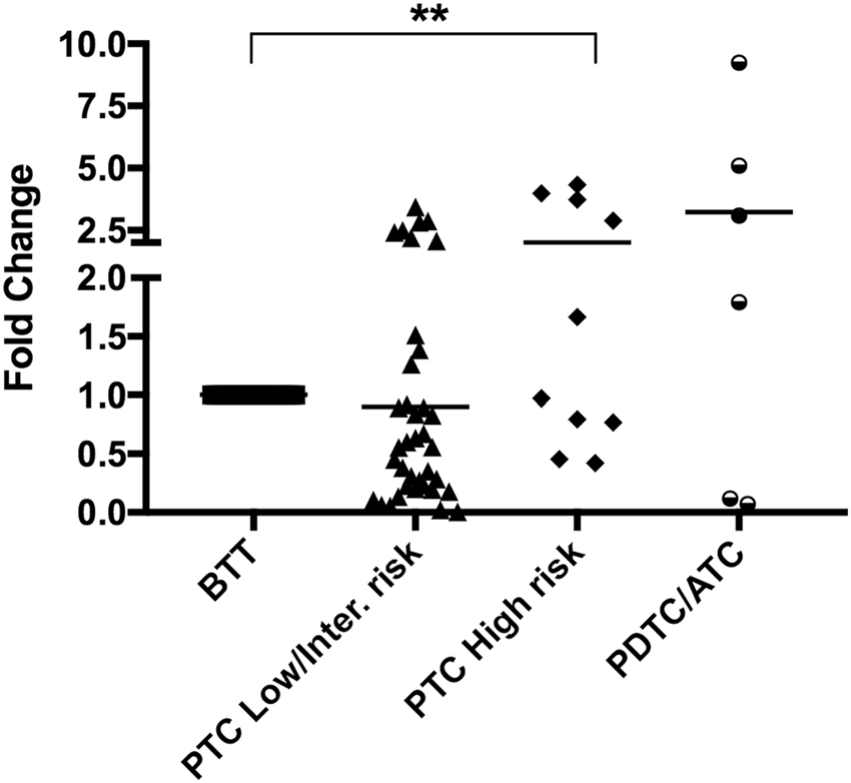
Analysis of the low density lipoprotein receptor (LDLR) gene expression by qRT-PCR in human thyroid tumors. BTT (*n* = 26), low/intermediate-risk PTC (*n* = 37), high-risk PTC (*n* = 10), PDTC/ATC (*n* = 6). Endogenous expression of the GAPDH gene has been used to normalize the data and BTT as calibrator. Statistical analysis: ANOVA test plus Tukey’s post-test (**p < 0.01).

The gene expression of the LDLR (specific receptor-mediated endocytosis of LDL) was upregulated in the PTC tumors with greater malignancy (high-risk PTC and PDTC/ATC) compared with the tumors with a minor degree of malignancy (low/intermediate-risk PTC and BTT; (Fig. 1). However, the tumorigenic-free cholesterol levels, esterified cholesterol, and triglycerides did not differ among all the groups analyzed (Supplementary Fig. S1). Moreover, the LDL uptake analysis in primary cell cultures displayed that the LDL-derived cholesterol uptake in the PDTC tumors was higher (13 ± 1.4%) than that of high-risk PTC and BTT tumorigenic cells (8.9 ± 0.3% and 8.4 ± 0.1%, respectively, *p* ≤ 0.01 *vs* PDTC) (Supplementary Fig. S2).

### HMGCR and CYP7B1 are strongly downregulated in PDTC and ATC tumors compared with BTT

**Figure 2:**
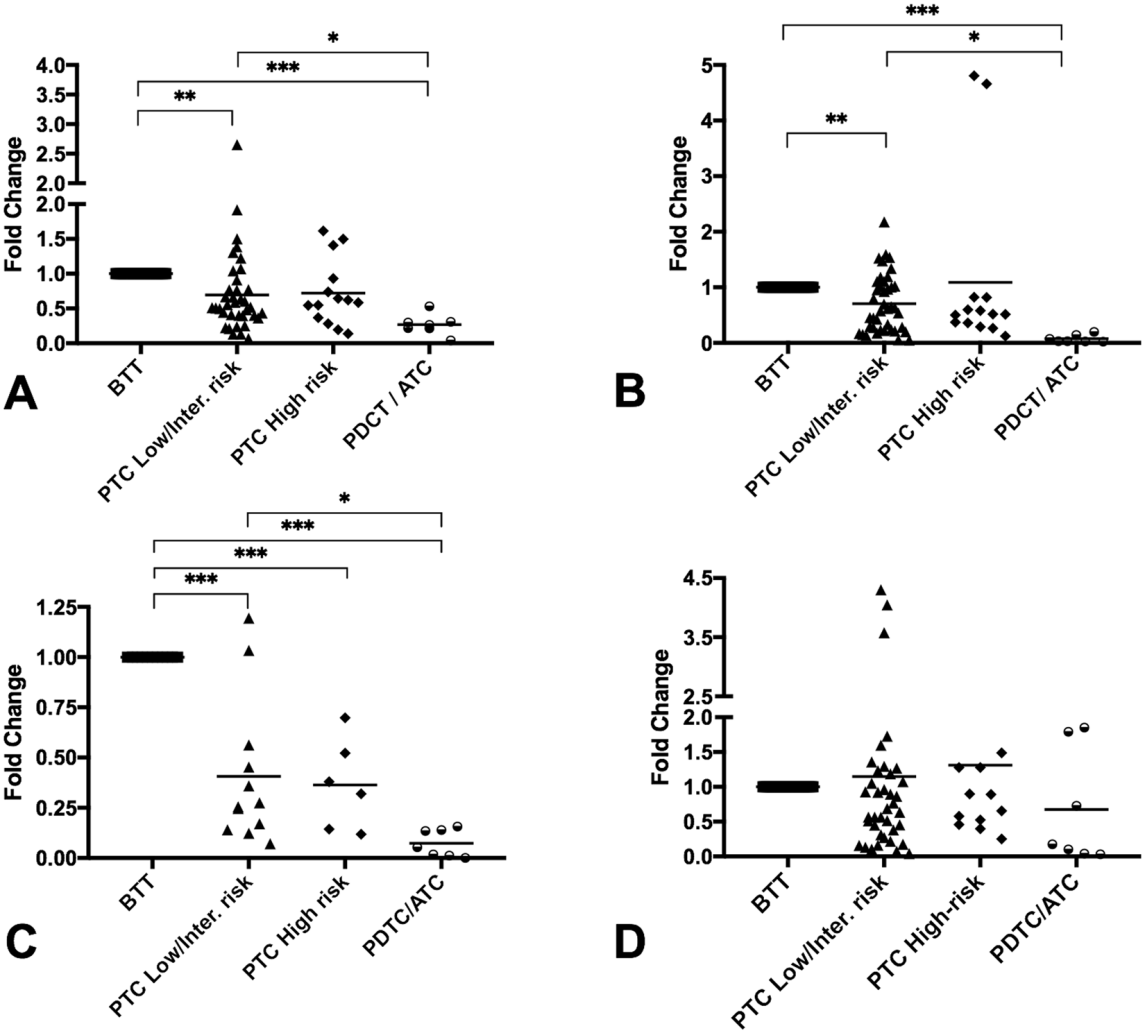
Analysis of the gene expressions by qRT-PCR in human thyroid tumors. (**A**) *HMGR* gene expression, (**B**) *CYP7B1* gene expression, (**C**) *LXR* gene expression, (**D**) *CYP27A1* gene expression. The patient cohort was divided into the following groups: BTT (*n* = 32), low/intermediate-risk PTC (*n* = 37), high-risk PTC (*n* = 12), and PDTC/ATC (*n* = 7). Endogenous expression of the *GADPH* gene was used to normalize the data, and BTT was used as calibrator tissue. Statistical analysis: ANOVA test plus Tukey’s post-test (*p < 0.05, **p < 0.01, ***p < 0.001).

The gene expression analysis of genes related with cholesterol metabolism and the oxysterol 27-HC in TC tumors are shown in Fig. 2; the expression of the HMGCR gene was downregulated in all types of malignant tumors compared to benign tumors, being higher in the PDTC/ATC (*p* < 0.001). Moreover, the differences were also significant when less aggressive PTC tumors or more indolent PTC tumors (low/intermediate risk) were compared with the aggressive PDTC and ATC tumors (*p* < 0.05, Fig. 2A). However, no differences were found between PTC with different degrees of malignancy. A similar expression profile was observed in the *CYP7B1* gene, which was also downregulated in all tumors, especially dedifferentiated PDTC/ATC (*p* < 0.001). The comparison between the malignant tumors exhibited a significant difference between the PTC (low/intermediate risk) and PDTC/ATC samples (*p* < 0.01; Fig. 2B). The gene expression analysis of the *LXR alpha* gene also displayed a similar gene expression pattern, although the downregulation detected was higher and significant in all groups analyzed (Fig. 2C). In contrast, the *CYP27A1* gene expression did not differ significantly among the groups (Fig. 2D).

The analysis of 24-Dehydrocholesterol reductase (24DHCR) and the scavenger receptor class B type 1 (SCARB1) genes showed that both genes tended to be downregulated when compared the most aggressive tumors with BTT group, although these changes were not significantly different (Supplementary Table 1).

**Figure 3:**
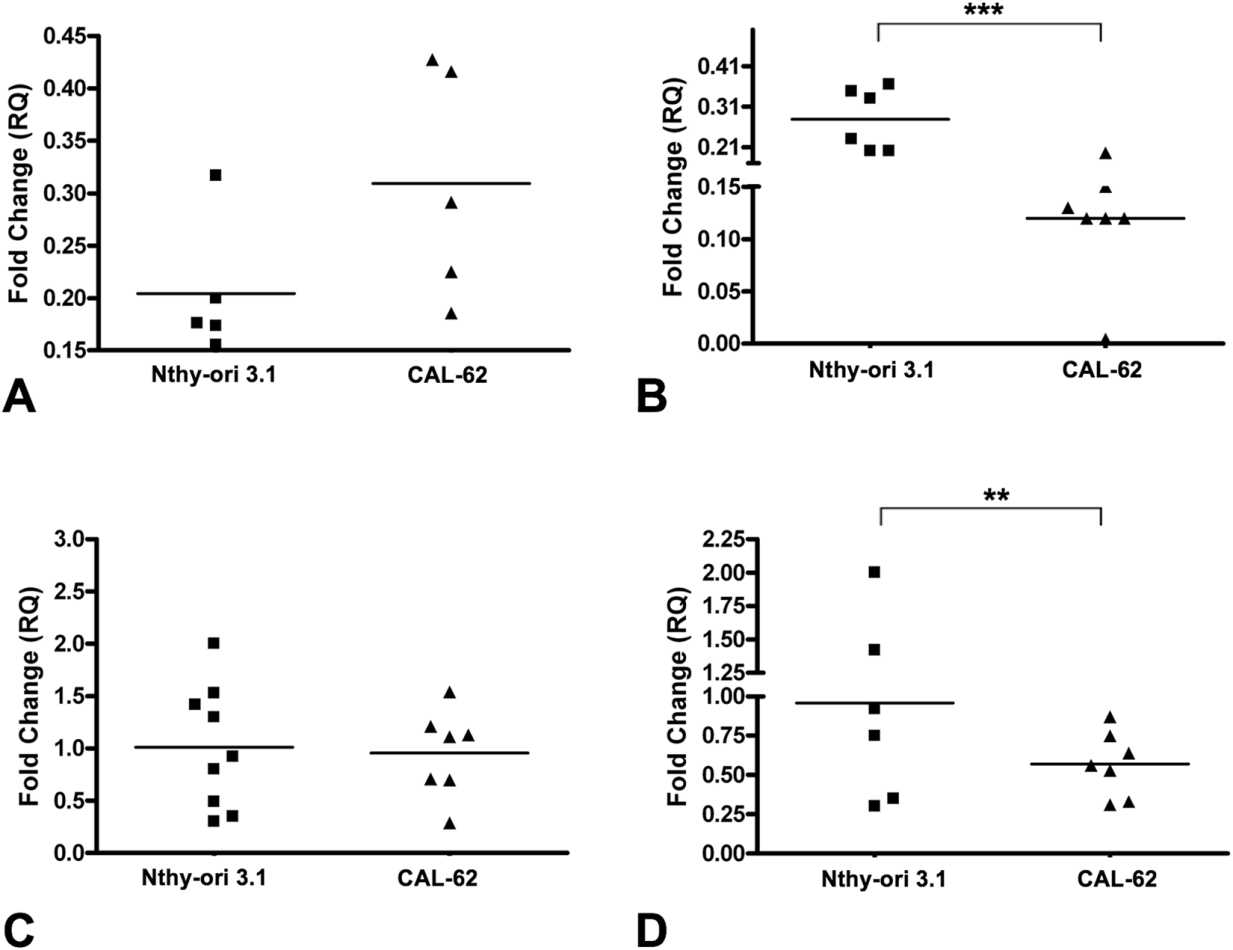
Analysis of the gene expression by qRT-PCR in thyroid cell lines (Nthy-ori 3.1 and CAL-62). (**A**) LDLR, (**B**) HMGR, (**C**) CYP27A1, and (**D**) CYP7B1. Endogenous expression of the GADPH gene was used to normalize the data. Statistical analysis: Student t-test (**p < 0.01, ***p < 0.001).

To investigate whether LDL can produce similar effects *in vitro* to those found *in vivo*, two thyroid human cell lines with different genotypes (Nthy-ori 3.1 and CAL-62) were exposed to human LDL and the gene expression profile was analyzed. The comparison of both cell lines did not reveal significant changes in LDLR and CYP27A1 gene expression (Fig. 3A,C). However, a downregulation in HMGCR and CYP7B1 was detected in CAL-62 in comparison with Nthy-ori 3.1 (Fig. 3B,D).

### PDTC/ATC tumors show higher levels of 27-hydroxycholesterol

**Figure 4:**
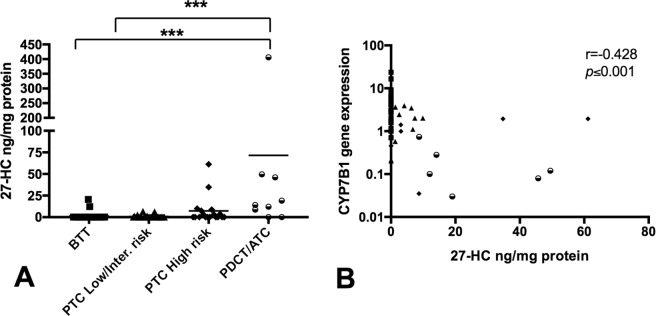
27-hydroxycholesterol (27-HC) content and their relationship with the CYP7B1 gene expression in thyroid tumor tissue extracts. (**A**) The analysis of 27-HC was measured in 82 patients (n = 97 thyroid tissue samples) corresponding to BTT (n = 40), low/intermediate-risk PTC (n = 30), high-risk PTC (n = 18) and PDTC/ATC (n = 9). The results represent the mean ± SD of the measurements of individual thyroid tissue. (ANOVA) using the Kruskal–Wallis test and Dunn’s post-test (***p < 0.001). (**B**) A correlation analysis was done in 73 samples in which 27-HC and CYP7B1 expression were analyzed in the same sample (27 BTT, 29 low/intermediate-risk PTC, 11 high-risk PTC and 6 PDTC/ATC samples). The graph illustrates the negative correlation between the CYP7B1 gene expression (2^−ΔΔCT^) and 27-hydroxycholesterol (27-HC) levels. CYP7B1 gene expression is showed log-transformed. R (−0.428), Pearson’s correlation coefficient; p ≤ 0.001.

The percentage of tumors with detectable levels of 27-HC was higher in the malignant tumors analyzed, with 20% in the low/intermediate-risk PTC samples (6 positive of the 30 total samples), with a mean of 3.73 ng/mg protein; 44% in the high-risk PTC samples (8 positive of 18 total samples), with a mean of 10.07 ng/mg protein; and 89% in the PDTC/ATC (8 positive of 9 total samples), with a mean of 89.42 ng/mg protein. In contrast, the percentage of detection in the BTT samples was 5% (2 of 40 total samples), with a mean of 16.38 ng/mg protein (Fig. 4A). Moreover, we observed a negative correlation between the CYP7B1 gene expression and the 27-HC concentration, showing higher levels of this sterol in the most aggressive samples analyzed (Fig. 4B).

### LDL cholesterol promotes proliferation and migration in anaplastic thyroid cell lines without changing in MAPK, PI3K and mTOR signaling pathways

**Figure 5:**
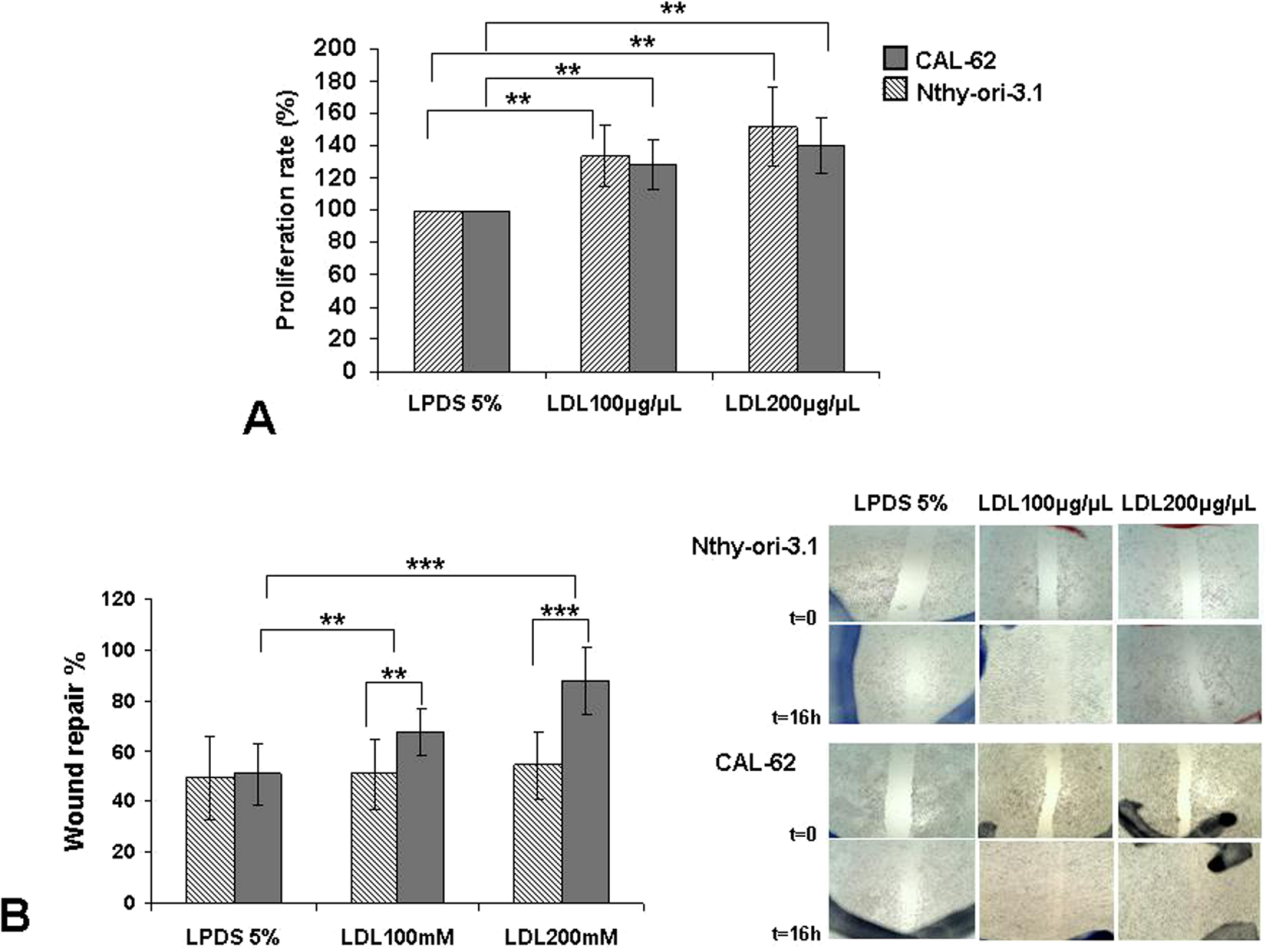
Exogenous administration of human low-density lipoprotein (LDL) in thyroid cell lines. (**A**) Percentage of cellular proliferation of the Nthy-ori 3.1 and CAL-62 cell lines. Both were treated for 24 h with LDL cholesterol (100 μg/mL and 200 μg/mL) compared with control cells maintained in basal conditions (5% LPDS). (**B**) Monolayer wound-induced migration assay. A line was scratched with a 200-µm plastic pipette tip in CAL-62 and Nthy-ori 3.1 cell lines; cultures were treated for 24 h with LDL cholesterol (100 mg/mL and 200 mg/mL). After 16 h, cells that had migrated to the wounded areas were photographed under a microscope for quantification of cell migration. Images are representative of three separate experiments. Quantitative analysis of wound-induced migration assay compared with control cells maintained in basal conditions (5% LPDS). The results are presented as mean ± SEM of eight experiments done in duplicate. The Kruskal–Wallis test was represented as a ± box plot, *n* = 8 separate experiments (**p < 0.01, ***p < 0.001).

The Nthy-ori 3.1 cell line, derived from normal human primary thyroid follicular epithelial cells, was immortalized with a plasmid containing an origin-defective SV40 genome (SV-ori). The CAL-62 cell line, derived from a human thyroid anaplastic carcinoma (ATC), is an epithelial-like cells stabilized in culture. These cells do not show thyroglobulin expression, as well as, do not present any BRAF mutation^29^. LDL promoted a dose-related response in cellular proliferation in both Nthy-ori 3.1 and CAL-62 (p < 0.01), without significance between them (Fig. 5A). Nevertheless, the evaluation of wound healing capacity showed that LDL cholesterol promoted cell mobility in the CAL-62 cell line in a dose-related response; in contrast, LDL cholesterol did not induce significant changes in the cell mobility in the Nthy-ori 3.1 cells (Fig. 5B). The concentration of 27-HC measured in the LDL treated cells and non treated cell was 3.73 ± 1.69 ng 27HC/mg prot and 0.37 ± 0.53 ng 27HC/mg prot, respectively (p = 0.01), in CAL-62 cell line. However, the concentration of 27-HC in the Nthy-ori 3.1 cells treated with LDL was lower in comparison with CAL-62 cell line (0.7 ± 0.25 ng 27-HC/mg prot) and it was undetectable in non treated cell.

Finally, the effect of the LDL in the MAPK, PI3K and mTOR signaling was also investigated. A slight dose-dependent decrease of the P-Akt and ERK was found in both cell lines (Supplementary Fig. S3).

### CYP7B1 overexpression and CYP27A1 silencing can modulate cellular proliferation rate

**Figure 6:**
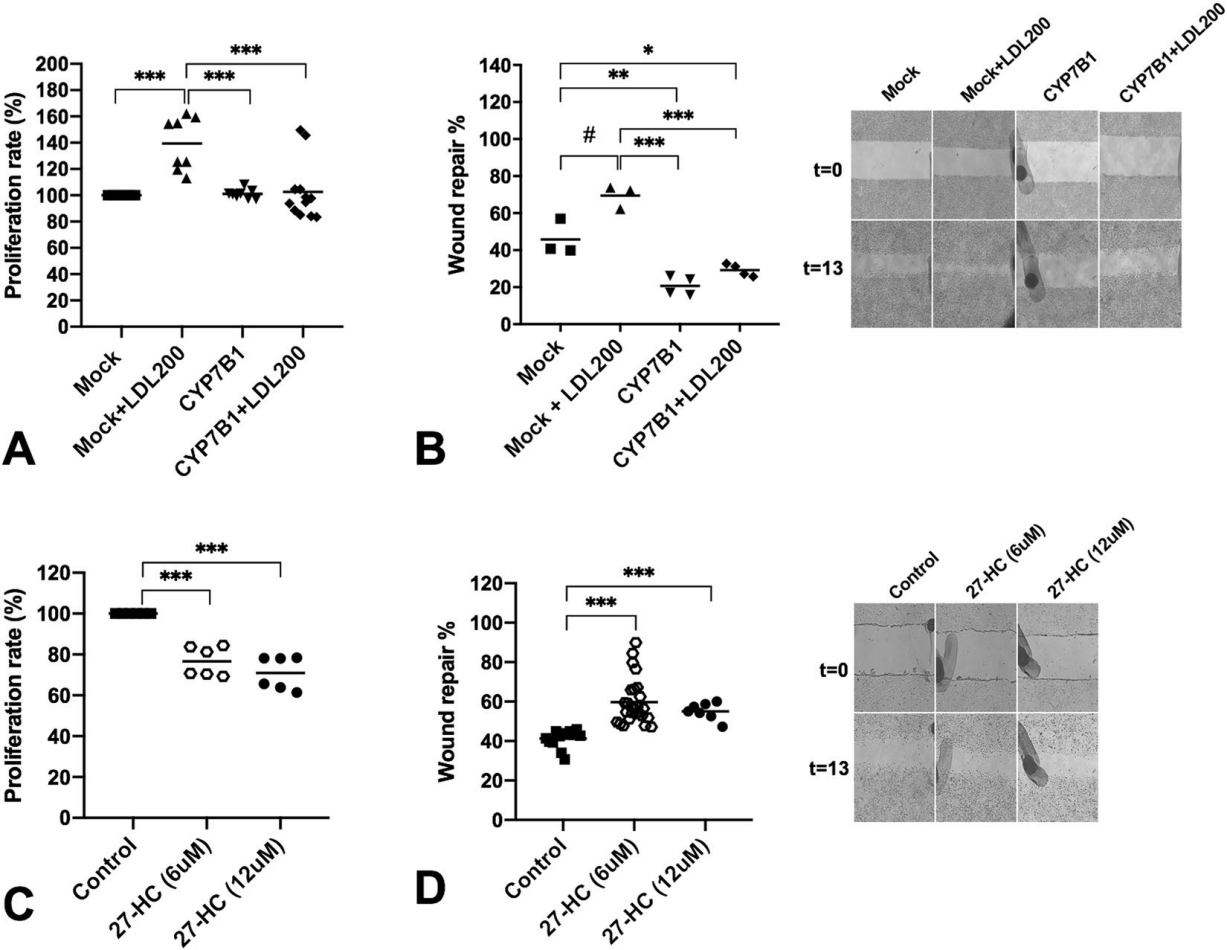
Overexpression of CYP7B1 gene in CAL-62 cells and exogenous administration of 27-HC in Nthy-ori 3.1 cell. (**A**) Percentage of cellular proliferation of the CAL-62 cells overexpressing CYP7B1 gene. Cells were treated for 24 h with or without LDL cholesterol (200 μg/mL) compared with control cells maintained in basal conditions (5% FBS). (**B**) Monolayer wound-induced migration assay in CAL-62 cells overexpressing CYP7B1 gene were scratched with a 200-µm plastic pipette tip and treated for 24 h with or without LDL cholesterol (200 μg/mL). After 13 h the wounded areas were photographed under a microscope for quantification of cell migration. Representative images of experiments. (**C**) Percentage of cellular proliferation of the Nthy-ori 3.1 cells treated with 27-HC at 6 and 12 μM for 48 h. (**D**) Monolayer wound-induced migration assay in Nthy-ori 3.1 cells gene were scratched with a 200-µm plastic pipette tip and treated for 48 h with 27-HC at 6 and 12 μM. After 13 h the wounded areas were photographed under a microscope for quantification of cell migration. Statistical analysis: ANOVA test plus Tukey’s post-test (*^,#^*p* < 0.05, **p < 0.01, ***p < 0.001).

The overexpression of CYP7B1 in CAL-62 cell line treated with LDL arrested growth (*p* = 0.001) and decreased cellular migration with (*p* = 0.02) and without LDL treatment (*p* = 0.02) (Fig. 6A,B); nonetheless, Nthy-ori 3.1 cell line treated with 27-HC at 6 and 12 µM decreased the proliferation rate in both concentrations (*p* = 0.001), but promoted the migration rate (p = 0.001) without evidence of a dose-related response (Fig. 6C,D).

Furthermore, downregulation of CYP27A1 completely blocked LDL-mediated cell proliferation (Supplementary Fig. S4). In contrast, DHEA did not promote proliferation in CAL-62 cell line, whereas a moderate arrest growth was detected in a doses dependent manner in Nthy-ori 3.1 cell line (Supplementary Fig. S5).

## Discussion

The role of cholesterol in cancer development is controversial; notwithstanding, several epidemiological studies have reported an association between serum cholesterol levels and a higher incidence of some types of solid tumors^18,26,30^. In this study, the patients bearing tumors with more aggressive behavior showed a significant decrease in serum LDL-cholesterol and apoB, without changing the HDL-cholesterol levels. These data are in consonance with different reports highlighting the importance of the uptake of systemic cholesterol as a mechanism modulating the tumoral process^31^. According to these data, we also detected overexpression of the LDLR in the subtypes of thyroid tumors with a more aggressive behavior. These data reinforce the hypothesis of these subtypes of tumors’ higher systemic cholesterol uptake capacity in comparison with benign tumors. There is evidence in tumors that has demonstrated the role of LDL-cholesterol on the cell migration and metastasis^3,32^. The promotion of the migration process after incubation with LDL cholesterol in cells could support the idea of cholesterol’s role in the tumor’s metastatic promotion. On the other hand, different signaling pathways have been proposed as a mediator in the control of development, progression, invasion and metastasis in thyroid cancer. Some of them, such as mitogen-activated protein kinase (MAPK), phosphoinositide 3-kinase (PI3K)/Akt, mTOR, has a strong relation with BRAF gene mutation, which are frequent in thyroid cancers^33^. The protein expression analyses did not show any change after LDL exposition in both CAL62 (wild-type BRAF and G12R KRAS mutation) and Nthy-ori 3.1 cell lines (no mutation has been reported). However, strong constitutive activation of these signaling transduction cascades under basal conditions were detected, indicating that the mechanisms by which LDL act are independent of these pathways. Additional cell lines harboring different oncogenic drivers should be investigated. In this context, SCARB1, which has been involved in tumor development^34,35^, tended to be downregulated when compared the most aggressive tumors with BTT group, although these changes were not significantly different. This result rather indicates that this receptor seems no to be associated with the tumor’s aggressiveness.

The regulation of the cholesterol metabolism in tumoral cells is complex. It is known that cancer cells have the capacity to increase intracholesterol via a feedback regulation mechanism of the HMGCR enzyme, which controls de novo cholesterol synthesis^36,37^. Importantly, the expression of this gene was downregulated in association with the degree of malignancies of the tumors. These changes may be related with similar free cholesterol levels found in the more aggressive tumors compared with those of benign tumors. It is noteworthy that the analyses in normal and ATC cell lines revealed a gene expression pattern like that found in the thyroid tumors from the patients. In contrast, one of the metabolites of cholesterol with greater relevance in both inflammatory and tumor processes is 27-HC, the most abundant oxysterol in the systemic circulation. In 2013, Nelson *et al*. demonstrated that 27-HC acted as an estrogen receptor agonist in breast cancer, inducing tumor growth and metastasis. These effects were reproduced in mice by overexpressing the CYP27A1 gene, which regulates 27-HC synthesis^18^. In contrast, decreased expression of CYP7B1 triggers accumulation of 27-HC, and CYP7B1 was downregulated in breast cancer compared with normal breast tissue^19,20^. In line with these findings, CYP7B1 was strongly downregulated in the aggressive tumor tissues (PTC high risk and PDTC/ATC) in relation to the benign tumors, in close association with the higher concentration of 27-HC. These data indicate that the accumulation of 27-HC in the thyroid cells may be promoting the development and progression of TC. In fact, downregulation of CYP7B1 was also detected in the anaplastic cell line (CAL-62) and its overexpression reduced cell growth and migration. Furthermore, downregulation of CYP27A1 completely blocked LDL-mediated cell proliferation. However, 27-HC by itself only promoted cell migration in non tumoral cells. We also treated the cells with DHEA, which may be hydroxilated by CYP7B1^38^ and it did not produce any effect in the cells with anaplastic phenotype. Nevertheless, in non-cancer cell (Nthy-ori 3.1 cells) we found a doses dependent decline in cell proliferation, as occurred with 27-HC. These results rather indicate a dual effect of 27-HC in cells with aggressive behaviors in contrast with immortalized cells with non tumoral phenotype. It should be noted that 27-HC increased ROS levels and reduced the antioxidant defense system levels in non pathological astrocytes, thereby affecting cell viability^39^. In addition, 27-HC may downregulate the expression of the nuclear factor E2-related factor 2 signaling pathways^40^. Moreover, in immortalized retinal pigment epithelial cell line (RPE cells), 27-HC also caused glutathione depletion, ROS generation, inflammation and apoptotic-mediated cell death^41^.

It should be noted that 27-HC is also involved in the regulation of the nuclear receptor LXR expression. This gene, which controls the cell cycle progression, is also implicated in the mechanism for controlling the metastasis of the tumors of epithelial lineage^42,43^. Moreover, the LXR gene controls the mechanism to promote liver LDLR degradation and cholesterol conversion to bile acids via CYP7A1^44,45^. The downregulation of LXR could also be directly affecting the LDLR levels in the aggressive tumors. The DHCR24, which catalyzes the conversion of desmosterol into cholesterol and is considered a tumoral biomarker involved in proliferation, adhesion, cell migration and apoptosis^46,47^, tended to be downregulated when compared the most aggressive tumors with BTT group, although these changes were not significantly different. Further investigation is needed to establish the role of desmosterol on thyroid cancer.

The expression of ATP Binding Cassette Subfamily A Member 1 (ABCA1), the main cholesterol efflux transporter from cells to HDL, was also determined. ABCA1 was upregulated in the most aggressive samples although this change was not significant in the PDTC group (Supplementary Table 1). We have recently reviewed the potential role of ABCA1 on tumor development. ABCA1 regulates plasma membrane cholesterol content and fluidity which are critical determinants of its metastatic capacity. However, ABCA1 also inhibits the development of tumors by inhibiting cellular proliferation thereby highlighting the dual role of ABCA1 in regulating proliferation and metastasis^48^. Further studies are needed to establish the role of ABCA1 on thyroid cancer.

Several studies have shown the relationship between the levels of 27-HC and the expression of estrogen receptors in the breast tumors, demonstrating that this oxysterol is a selective modulator of them, having a role as an agonist or antagonist in the function of the cellular type^18,19^. In relation to epithelial-type thyroid cancer, these have a higher incidence in females. This characteristic probably suggests its relationship with the expression of estrogen receptors in the follicular thyroid epithelium. There are not enough reported data identifying the expression of these sexual steroid receptors and their relationship with the neoplastic follicular cells^49^. We explored the expression of the estrogen receptors (alpha and beta) in a series of samples, and the results showed that both benign and malignant tumors express both types of receptors. However, their expression was observed in undifferentiated tumors (Supplementary Table S2). These data are consistent with those observed in breast cancer, where estrogen receptor (ER)-negative tumors tend to grow faster and have a worse prognosis, whereas a lack of ER isoforms β expression would lead to a decrease in apoptosis^50,51^. This result contrasted with the potential effect of 27-HC as an endogenous ER ligand that promotes ER-positive breast tumor growth^19^. In contrast, 27-HC treatment reduced cell growth in Nthy-ori3.1 cells (ERα/ß negative). This effect is in agreement with the reported in prostate cancer cells in which 27-HC reduced intracellular cholesterol accumulation independent of the androgen receptors status^52^. These results suggest that 27-HC could promote a negative feedback control of cholesterol biosynthesis in normal immortalized follicular cells. Studies of the potential effects of 27-HC on ER-independent tumorigenic processes are warranted.

**Figure 7:**
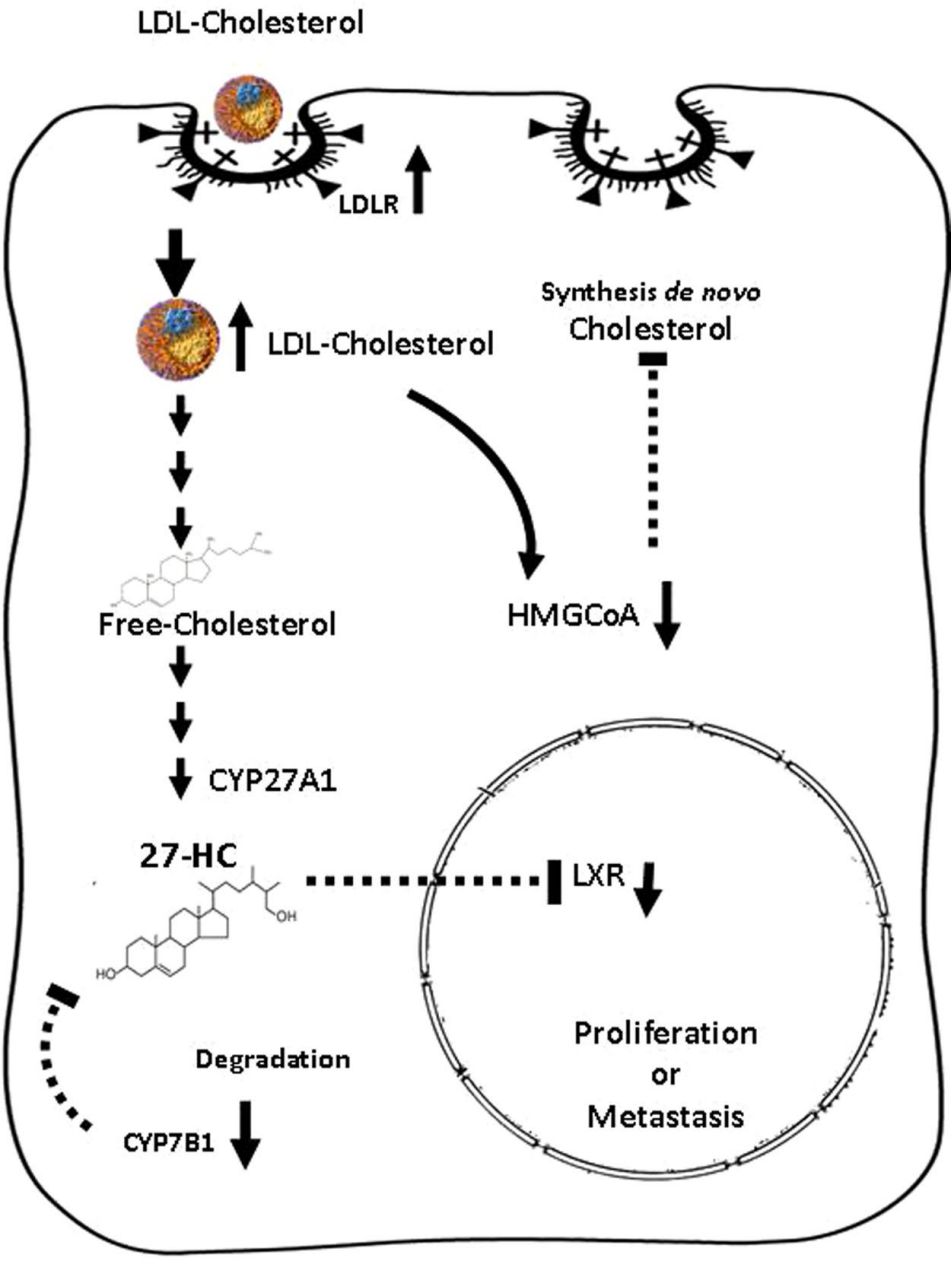
Schematic drawing of the hypothesis of the effect of uptake and intracellular pathways of LDL in the thyroid cells, based on our results. The LDL cholesterol molecules are internalized via endocytotic vesicles, transported and rapidly metabolized; they start to accumulate in the form of 27-HC due to the inhibition of their degradation in the aggressive forms of tumors. The oxysterol could diffuse into the nucleus region and interact with nuclear receptors and other molecular targets, promoting the proliferation or metastatic processes in the follicular cells. 3-hidroxi-3-metilglutaril-CoA (HMG-CoA) downregulation indicates a potential negative feedback in the *de novo* synthesis of cholesterol.

Of note, some experimental studies have shown the antineoplastic efficacy of statins but statins were associated with thyroid carcinoma in some clinical studies^53^. Furthermore, hepatic PCSK9 expression is modified in several diseases, including in thyroid cancer^54^: however, no large genetic studies and controlled cholesterol-lowering clinical trials have been carried out yet. In summary, our findings showed that cholesterol could be a key molecule in the thyroid carcinogenesis process, especially in terms of 27-OH, which may influence the development and progression of TC (Fig. 7). Therefore, targeting the transport of cholesterol and synthesis of 27-HC may be a therapeutic strategy for controlling TC development in the future. The causal association of cholesterol/27-HC and TC should be further investigated.

## Material and Methods

### Data sharing

The data, analytical methods, and study materials will be available to other researchers for purposes of reproducing the results or replicating the procedure upon reasonably request.

### Patients

This study included a cohort of patients (*n* = 89) with a diagnosis of TC who were recruited at Hospital de la Santa Creu i Sant Pau (HSCSP) in Barcelona, Spain. In these patients, the following clinical information was collected: sex, age, unhealthy habits, no dyslipidemic pathology or statin treatment, and baseline TSH values (0.5–5 mlU/L). The tumors were classified according to the histopathological diagnosis, as follows: benign thyroid tumor (BTT), well-differentiated malignancies (PTC), and advanced thyroid cancers which include poorly differentiated and anaplastic thyroid carcinoma (PDTC/ATC). The AJCC/UICC TNM staging system (TNM) was used to subdivide the patients into two groups (low/intermediate and high risk; NCCN Guidelines Version 1. 2018, Staging Thyroid Carcinoma; Table 2). This study was approved by the ethics committee at HSCSP, and all patients gave written informed consent to participate. All research was performed in accordance with relevant guidelines/regulations.

**Table 2:** Clinical Description of the study Cohort.

Patient Demographics	Age at Diagnosis	
SEX	52.12 ± 5.65 (16–84)	n = 89
Female	50.8 ± 16.5 (34–75)	n = 77
Male	53.5 ± 14.7 (16–84)	n = 12
**HISTOLOGY PATTERN**		
Benign thyroid tumor (BTT)	52.92 ± 13.35 (29–81)	n = 27
Papillary thyroid carcinoma (PTC)	49.8 ± 16.4 (16–72)	n = 55
Low/intermediate Risk		n = 43
TNM staging		
I		n = 26
II		n = 17
High Risk	44.23 ± 19.6 (29–84)	
TNM staging		n = 12
III		n = 7
Advanced Thyroid Cancers:	61.5 ± 15.75 (34–82)	
PDTC		n = 5
Anaplastic thyroid carcinoma (ATC).		n = 2

The age is represented as the mean ± standard deviation (minimum age–maximum age). Total subjects included in the study were classified, the AJCC/UICC TNM staging system (TNM) was used to subdivide the patients into two groups (low/intermediate and high risk; NCCN Guidelines Version.1 2018.

### Human tissue and serum samples

Surgical snap-frozen specimens and serum were obtained from patients recruited from January 2009 at HSCSP, as described above. These belonged to the sample collection registered at Instituto de Salut Carlos III (Spain; *C.000281*).

### Lipid, lipoprotein, and apolipoprotein determinations

Serum lipid analyses were determined enzymatically using commercial kits adapted to a COBAS c501 autoanalyzer (Roche Diagnostics, Rotkreuz, Switzerland)^55^. High-density lipoprotein (HDL) cholesterol was measured in apoB-depleted serum obtained after precipitation with phosphotungstic acid and magnesium ions (Roche Diagnostics). The low-density lipoprotein (LDL) cholesterol was determined by the Friedewald formula^56^. The apoA-I and apoB concentrations were determined using nephelometric commercial kits (Roche Diagnostics)^55^.

### Thyroid 27-hydroxycholesterol (27-HC) levels

Frozen thyroid samples (n = 66) included in optimal cutting temperature (OCT) compound (Tissue-Tek, Sakura Europe, Alphen den Rijn, Netherlands) were sectioned and homogenized in 1 M NaOH. Protein concentration was measured using the BCA protein assay kit (Thermo Scientific S.L.U., Spain). Lipids were then extracted as described above, resuspended in 100 μl of methanol, and transferred to a vial with 6 μl of deuterium-labeled internal standard. The 27-HC levels were measured using high-performance liquid chromatography tandem mass spectrometry (HPLC-MS/MS)^57^.

### Human lipoproteins isolation: Low-density lipoprotein (LDL) and high-density lipoprotein (HDL)

Human LDL (1.019–1.063 kg/L) and HDL (1.063–1.21 kg/L) were isolated by sequential ultracentrifugation of fasting plasma. Lipoprotein-depleted serum (LPDS) was prepared from fetal bovine serum (FBS) or charcoal/dextran-treated FBS (Gibco BRL, Life Technologies) by ultracentrifugation at a density > 1.21 kg/L.

### Primary cell cultures and cellular low-density lipoprotein (LDL) cholesterol uptake

Human thyroid tissue obtained from intraoperative pieces were dissected in the Department of Pathology, washed with PBS Buffer, minced into small fragments and digested with collagenase type II (1 mg/mL, Gibco Invitrogen) in stir at 37 C for 1–3 h. The reaction was stopped by adding 10 mL of culture medium at room temperature and the digested tissues were centrifuged 1000 g for 10 min and washed two times in PBS Buffer salt solution. The cellular pellets were suspended with DMEM-F12 medium, transferred in cellular plates, and the medium was changed every two days. The cultures were maintained only six days. Finally, the cells were trypsinized, counted using an automated cell counter (TC10 Bio-Rad) and cell blocks with 10^5^–10^6^ cells were prepared by plasma-thrombin method. In order to check cell thyroid purity, thyroglobulin immunostaining with sections from cell blocks was done by peroxidase-anti-peroxidase (PAP) technique.

LDL particles (1.019–1.063 kg/L) were isolated by sequential ultracentrifugation at 100,000 g for 24 h from normolipidemic human plasma obtained in EDTA-containing vacutainer tubes and radiolabeled with [1,2-^3^H] cholesteryl oleate (Perkin-Elmer), as previously reported^58^. Subsequently, radiolabeled LDL (0.45 μCi [1,2-^3^H] cholesterol oleate/50 µg LDL-apoB) was dialyzed against phosphate-buffered saline (PBS) by gel filtration chromatography and incubated with 10^5^ cells in DMEN-F12 supplemented with 5% LPDS for 16 h. Then, the medium was collected and centrifuged at 10,000 g for 10 min, and the cells were lysed with 0.1 M NaOH. The radioactivity was determined in the medium and cell lysates. [1, 2-^3^H] LDL cholesterol uptake was expressed as the percentage of the radioactivity collected in the cells relative to the sum of radioactivity in cells and medium per µg of protein per cell.

### Total RNA isolation and first strand cDNA synthesis

Total RNA was isolated using the TRIZOL reagent according to the manufacturer’s instructions (Invitrogen, Carlsbad, CA, USA). One microgram of the total RNA was reverse transcribed using a transcriptor first-strand cDNA synthesis kit (Roche Applied Science, Penzberg, Germany), and the cDNA samples were stored at –20 °C for use as a template in real-time polymerase chain reaction (PCR) analysis.

### Gene expression profile

The gene expression profiles were analyzed in an ABI PRISM 7900HF Sequence Detection System, using a predesigned and labeled primer/probe set (Assays-on-Demand™ Gene Expression Assay, Applied Biosystems, Foster City, CA, USA). The Taqman qPCR primers (Applied Biosystems) used were as follows: CYP27A1 (Hs01017992_m1), CYP7B1 (Hs01046431_m1), HMGR (Hs00168352_m1), LDL receptor (LDLR; Hs01092524_m1), NR1H3 (Hs00172885_m1), SCARB1 (Hs00969827_m1), DHCR24 (Hs00207388_m1) and ABCA1 (Hs01059137_m1). All the reactions were performed with 100 ng of cDNA in a total volume of 50 μl of TaqMan® Universal PCR Master Mix (Applied Biosystems), and the relative expression levels for each gene were calculated using the 2- ddCt method, with SDS2.3 and Data Assist V2.1 software (Applied Biosystems). The samples were analyzed in duplicate, and RNA from BTT was used as a group calibrator; negative controls were also included in all the reactions.

### Human thyroid cell lines and LDL, 27-HC and DHEA treatments

The Nthy-ori 3.1 cell line, derived from normal human primary thyroid follicular epithelial cells, was immortalized with a plasmid containing an origin-defective SV40 genome (SV-ori). This cell line was provided by Dr Pilar Santisteban (CSIC, Madrid), and the CAL-62 cell line, derived from a human thyroid anaplastic carcinoma (ATC), has a epithelial-like cells stabilized in culture. The cells do not show thyroglobulin expression and the most relevant cytogenetic data showed a gain of chromosome 20, with a translocation (14q), breakpoints of centrometric chromatine without presenting any BRAF mutation^29^. CAL-62 cell line was provided by Leibniz-Institut DSMZ GmbH (ACC 448). Nthy-ori 3.1 cell lines was cultured in RPMI 1640 (w/L-glutamine) and CAL-62 in Dulbecco’s modified Eagle’s medium (DMEM; Gibco, Invitrogen); both media were supplemented with 10% FBS and 2% streptomycin/penicillin. For treatment, 4,000 cells/well were seeded in 96-well microplates, and exogenous LDL obtained from human serum was added to the cultures at 200 μg/mL for 72 h, 27-HC at 6 and 12 μM for 48 h. For DHEA treatments (SKU 7000087 P) (Avanti, Polar Lipids, INC.), DHEA at 40, 80 and 160 uM was also added in both cells lines for 48 h.

To determine the cell viability rate, we used the thiazolyl blue tetrazolium bromide metabolic assay (MTT, Sigma, St. Louis, MO, USA). Absorbance was determined at 560 nm with a microplate reader (xMark, BIORAD). All conditions were performed in five replicates. Each experiment was repeated at least three times.

To determine cell migration, both cell lines (Nthy-ori 3.1 and CAL-62) were plated at high densities and grown to confluence at 90% O/N. Cells were scratched with a pipette tip (10 µl) and washed several times to remove the cellular debris.

The wounds were photographed in an inverted microscope at 0 h (t = 0), and this was done again in the same area after 16 h of cell incubation at 37 °C. The cultures were allowed to incubate in serum-free medium. Image J software was used to analyze the photographs. Here, the percentage of wound healing was determined based on three measurements of the wound area; each result was the mean of three independent experiments.

### Transient transfection assay

#### Overexpression of CYP7B1 gene in CAL-62 cell line

Transfection in CAL-62 cell line was performed by JetPrime™ (Polyplus transfection) according to the manufacturer’s instructions. Approximately 10,000 cells were plated in 96-well plates one day prior to transfection. The cells were transiently transfected with either 0.1 nM pre-designed CYP7B1 (NM_004820.3) DNA ORF clone or control (pcDNA3.1^+^/C-(K)DYK) purchased from GeneScript (OHu27221) and treated with LDL 200 µM or without LDL as a control. After incubation for 48 h, the transfected cells were tested for MTT assay. For wound-healing assays, 50 × 10^3^ cells were plated in 4-well plates, transiently transfected with the same concentration described above and the wound-healing assays were performed as described below. Each assay was performed in triplicate in at least three independent experiments.

### Downregulation of CYP27A1 gene in Nthy-ori 3.1 cell line

siRNA transfection in Nthy-ori 3.1 cell line was performed by JetPrime™ (Polyplus transfection) according to the manufacturer’s instructions. Approximately 4,000 cells were plated in 96-well plates one day prior to transfection. The following day, the cells were transiently transfected with either 5 nM pre-designed short interfering RNA (siRNA **CYP27A1**) (ID106239) (Ambion, Life Technologies) or control siRNA purchased from Santa Cruz Biotechnology, Inc. (sc-37007). The transfected cells were treated with LDL 200 µM or without LDL as a control. After incubation for 48 h, the transfected cells were tested for MTT assay. Each assay was performed in triplicate in at least three independent experiments.

### Statistical analysis

Gene expression analysis was performed using the comparative CT (ddCt) method by DataAssist™ Software, and the results were adjusted as the *p*-value using the Benjamini–Hochberg false discovery rate (FDR). Genes showing an FDR <0.15 and more than twofold difference in absolute values for both comparisons were considered differentially expressed. One-way analysis of variance (ANOVA) with a Tukey’s multiple comparison post-test or Kruskal–Wallis multiple comparison with Dunn’s post-test was used to compare differences among the groups. Correlations between variables were analyzed using Pearson’s correlation analysis. GraphPad Prism software V5.0 was employed, and values of *p* < 0.05 were considered statistically significant.

## Supplementary information

Supple figures and tables
