# Supplementary material for: Cholesterol and 27-hydroxycholesterol promote thyroid carcinoma aggressiveness
Source: Sci Rep. 2019 Jul 16;9:10260. doi: 10.1038/s41598-019-46727-2 (PMC6635382; doi:10.1038/s41598-019-46727-2)
Supplement: Supplementary file 1 — Supple figures and tables [file 41598_2019_46727_MOESM1_ESM.docx]

**Cholesterol and 27-hydroxycholesterol promote thyroid carcinoma aggressiveness**

Giovanna Revilla^2,3^, Monica de Pablo Pons^1,2^, Lucía Baila-Rueda^4,5^, Annabel García-León^2^, David Santos^2,6^, Ana Cenarro^5^, Marcelo Magalhaes^8^, R. M.ª Blanco^7^,Antonio Moral^2^,^9,10^, José Ignacio Pérez^9^, Gerard Sabé^2^, Cintia González^1,7^, Victoria Fuste^2,11^, Enrique Lerma^2,11^, Manuel dos Santos Faria^8^, Alberto de Leiva^2,7^, Rosa Corcoy^1,7,10^, Joan Carles Escolà-Gil^2,3,6*^,and Eugenia Mato^1,7*^

**Thyroid cholesterollevels**

Frozen thyroid tissues (*n* = 66) were homogenized in 1 M NaOH, and lipids were extracted with isopropyl alcohol-hexane 2:3 (v/v); after the addition of Na_2_SO_4_, the hexane phase was isolated and dried with N_2_. Lipid extracts were resuspended in chloroform, and free and esterified cholesterol was partitioned by thin layer chromatography. The chromatographic developing solution was heptane/diethylether/acetic acid (74:21:4, v/v/v). The spot corresponding to free and esterified cholesterol was quantified by densitometry against the standard of free cholesterol and cholesterol oleate using a computing densitometer^1^.

### Western Blot Analysis

Protein extracts from cell line CAL-62 and Nthy-ori 3.1 cell lines were obtained after homogenizingthe cells in a buffer containing 50 mm Tris-HCl (pH 7.5), 150 mm NaCl, 1% (v/v) Triton X-100, and a protease inhibitor mixture (Sigma). Proteins were quantified and 10 µg of total protein were separated by 12 or 7.5 % TGX Stain-Free polyacrylamide Starter kit (161-0174 Bio-Rad, Carson, CA), and transferred to PVDF membranes. The antibodies used to incubate the membranes were**:** Mouse Anti-ERK (pan ERK) Clone 16/ERK (pan ERK) (BD Bioscience610123) 1:10000, anti-Phospho-p44/42 MAPK (Erk1/2) (Thr202/Tyr204) (Cell Signaling 9101) 1:4000, anti-Akt (pan) (11E7) Rabbit mAb(Cell Signaling 4685) 1:2000, Anti-Phospho-Akt (Ser473) (D9E) XP^®^ Rabbit (Cell Signaling 4060)1:2000, Anti-mTOR (Cell Signaling 2972)1:4000, Anti-Phospho-mTOR (Ser2448) antibody (Cell Signaling 2971) 1:4000. Chemiluminescence was revealed using an Immun-Star WesternC Chemiluminescence Kit (170-5070, Bio-Rad, Carson, CA).Imaging and data analysis:TGX Stain-Free gels were activated for 1’ after SDS–electrophoresis, the images were captured by ChemiDOC^TM^XRS Gel Documentation Systems (Bio-Rad, Herculles, CA) ImageLab software (version 5.1, Bio-Rad, Richmond, CA). The data normalization analysis was performed with stain free^2^.

**Gene expression of estrogen receptors**

The expression of the estrogen receptors were performed by reversetranscriptase-polymerase chain reaction (*RT*-*PCR*) from 50ng of cDNA in a final volume of 25μl and specific primers. ERα: Forward5’*CAGGGGTGAAGTGGGGTCTGCTG*3’, Reverse 5’*TGCCTCCCCGTGATGTAAT*3’(762-935bp) and temperature annealing (65ºC),ERβ**:** Forward*5’CCCTGCTGTGATGAATTACAG*3’, Reverse 5’*CTTCTCTGTCTCCGCACAAG*3’ (552bp) and temperature annealing (61ºC). The amplification was done with a Taqhotstart (BioThermStar DNA Polymerase, Gene Craft GC-045-0500)for 35 cycles (95 C 10 min and 95 C 1 min, 65 Cor 61 C 1 min, 72 C 1 min)with a final extension of 10 min at 72 C. The PCR products were subjected to electrophoresis in 1% agarose gel.

**REFERENCES**

1. de Gonzalo-Calvo, D. *et al.*Intratumor cholesteryl ester accumulation is associated with human breast cancer proliferation and aggressive potential: a molecular and clinicopathological study. *BMC Cancer***15,** 460; doi: 10.1186/s12885-015-1469-5 (2015).

2. Gürtler, A. *et al.* Stain-free technology as a normalization tool in western blot analysis. *Anal Biochem.***433,** 105–11; doi:10.1016/j.ab.2012.10.010 (2013).


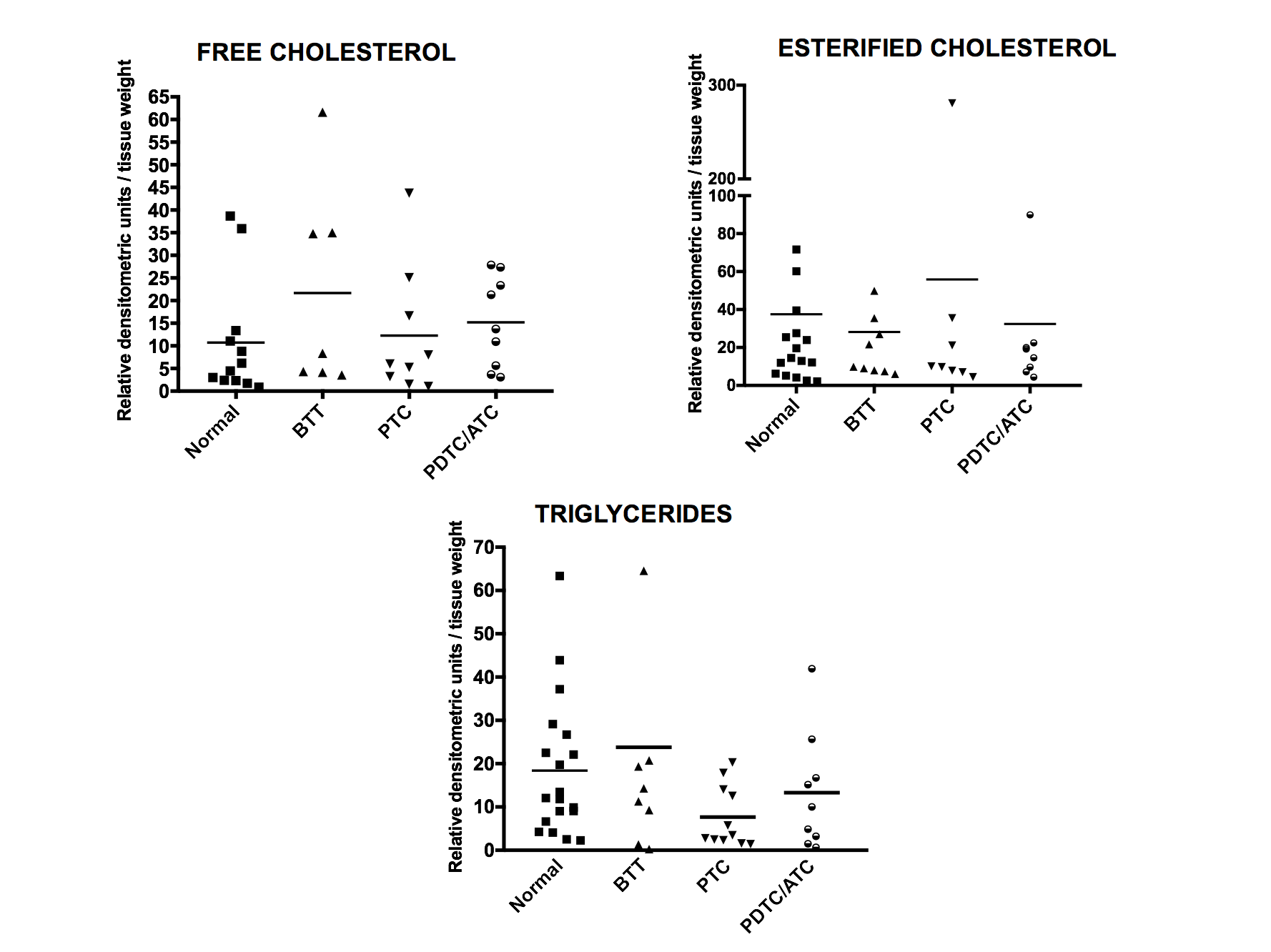


**Supplementary Figure S1.** Free Cholesterol, Esterified Cholesterol, and Triglycerides did not change in thyroid tumor tissue extracts and normal thyroid tissue.

Lipid extracts from BTT, PTC, PDTC,ATC and Normal Thyroid tissue (Normal) were determined by thin layer chromatography. Analysis of variance using ANOVA plus Tukey’s post-hoc test did no shown significant differences.


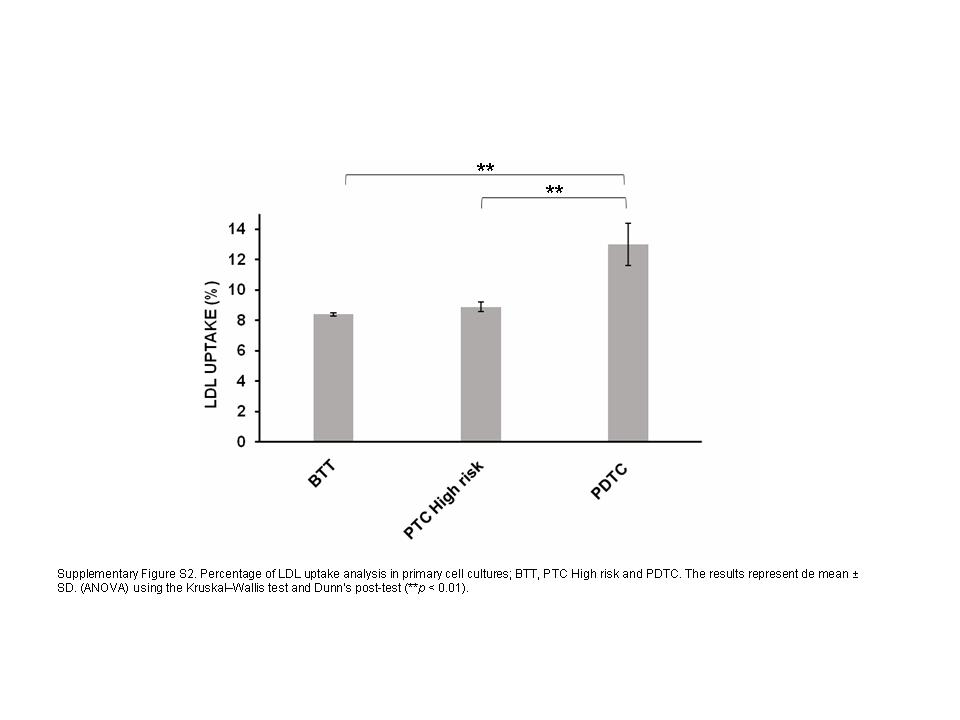


**Supplementary Figure S2**. Percentage of LDL uptake analysis in primary cell cultures; BTT, PTC High risk and PDTC. The results represent de mean ± SD. (ANOVA) using the Kruskal–Wallis test and Dunn’s post-test (***p*< 0.01).


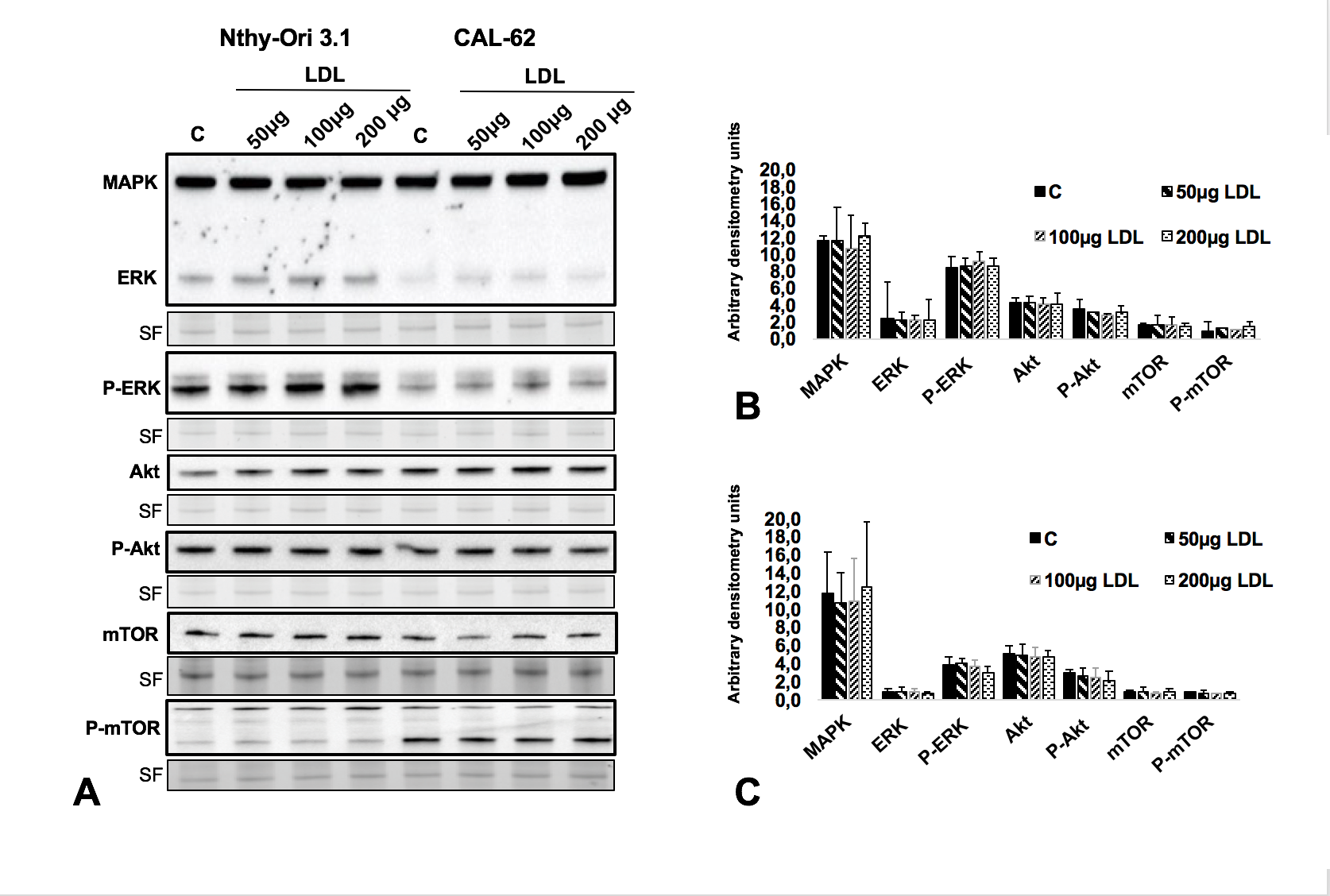


**Supplementary Figure S3.**LDL (50, 100 and 200 µM) incubated during 48h, does not promote changes in MAPK, PI3K and mTOR signaling pathways in both cells lines (CAL-62 and Nthy-ori 3.1). A) Representative Western blotof total proteinextractsfromCAL-62 and Nthy-ori 3.1 withantibodiesagainstAnti-ERK (pan ERK), anti-Phospho-p44/42 MAPK (Erk1/2)anti-Akt, Anti-Phospho-Akt, Anti-mTOR, Anti-Phospho-mTOR. **B-C)**Signals were normalized to stainfree (SF) gels and data presented asmean of integratedrelativedensityvaluefromNthy-ori 3.1 and CAL-62, respectively. Data areexpressed as mean ± SEM (*n* = 3).


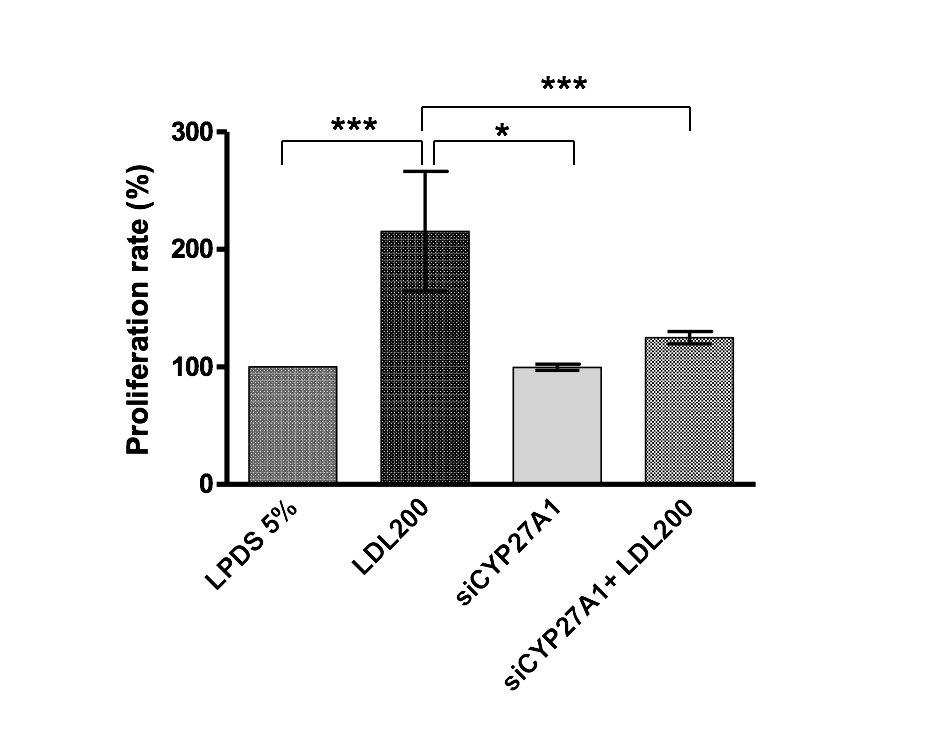


**Supplementary Figure S4.** Downregulation of CYP27A1 gene in Nthy-ori 3.1. Percentage of cellular proliferation of the Nthy-ori 3.1 cells with CYP27A1 downregulated. Cells were treated for 48 h with or without LDL cholesterol (200 μg/mL) compared with control cells maintained in basal conditions (5% LPDS). Statistical analysis: ANOVA test plus Tukey’s post-test (**p*<0.05, ****p*<0.001).


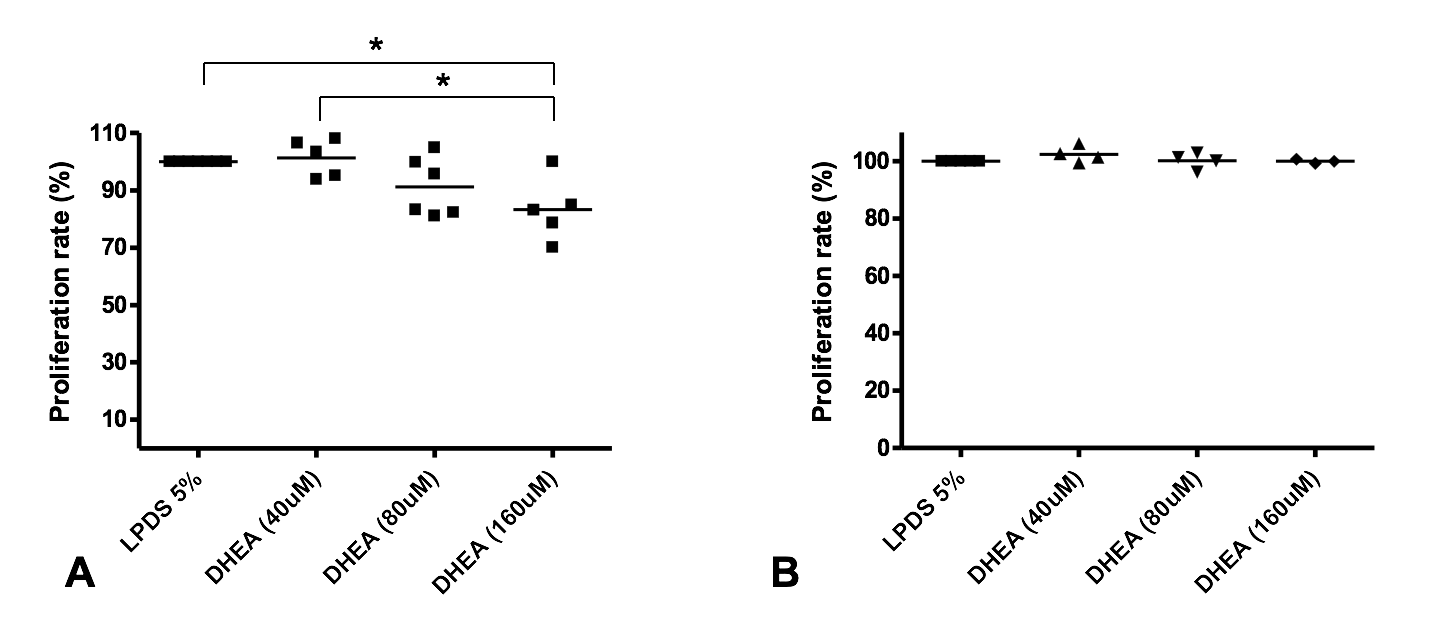


**Supplementary Figure S5.** Exogenous administration of DHEA in Nthy-ori 3.1 and CAL-62 cell lines. **A)** Percentage of cellular proliferation of the Nthy-ori 3.1 cells treated for 48 h with or without DHEA (40uM, 80uM and 160uM) compared with control cells maintained in basal conditions (5% LPDS). **B)** Percentage of cellular proliferation of the CAL-62 cells treated for 48 h with or without DHEA (40uM, 80uM and 160uM) compared with control cells maintained in basal conditions (5% LPDS). Statistical analysis: Kruskal-Wallis test and Dunn’s Comparison post-test (**p*< 0.05).

**Supplementary Table 1: Gene Expression of ATP-binding cassette transporter 1 (ABCA1), 24-Dehydrocholesterol Reductase (DHEA) and Scavenger Receptor Class B (SCARB1) Member genes by qRT-PCR**

|  |  | **GENE** | **PTC** |  | **PTC HIGH RISK** |  | **PDTC** |  |
| --- | --- | --- | --- | --- | --- | --- | --- | --- |
| **Gene Name** | **Gene Symbol** | **ID** | **(RQ)** | **(P-Value)** | **(RQ)** | **(P-Value)** | **(RQ)** | **(P-Value)** |
| **ATP Binding Cassette Subfamily A Member 1** | **ABCA1** | 19 | 5.3007 | 0.0008 | 7.9018 | 0.0377 | 7.1117 | 0.5044 |
| **24-dehydrocholesterol reductase** | **DHCR24** | 1718 | 0.6044 | 0.5329 | 0.6297 | 0.5681 | 0.2775 | 0.2513 |
| **Scavenger receptor class B member 1** | **SCARB1** | 949 | 0.4356 | 0.3813 | 0.5038 | 0.5681 | 0.3138 | 0.2513 |

### Differential gene expression was detected by qRT-PCR from human epithelial thyroid carcinoma, PTC (n=10), PTC HIGH RISK (n=16), PDTC/ATC (n=3). Data are presented as the fold change in target gene expression in tumours normalized to the BTT was the calibrator tissue (n=12) Statistical analysis: ANOVA test plus Tukey’s post-test.

**Supplementary Table 2: Gene Expression of Estrogen Receptor (ER)α and ERβ isoformsaccording to histologypattern**

|  |  | **BTT** | **PTC (low/**  **intermediate risk)** | **PTC (high risk)** | **PDTC/ATC** |
| --- | --- | --- | --- | --- | --- |
| **REα** | **Positive** | 24 (77.4%) | 35 (87.5%) | 10(66.7%) | 2 (23%)** |
|  | **Negative** | 7 (22.6%) | 5 (12.5%) | 5 (33.3%) | 7 (78%)** |
|  |  |  |  |  |  |
| **REβ** | **Positive** | 27 (87.1%) | 37 (92.5%) | 9 (60.0%) | 1 (11%)*** |
|  | **Negative** | 4 (12.9%) | 3 (7.5%) | 6 (40.0%) | 8 (89%)*** |

The analysis was measured in 89 patients (95 thyroid tissue samples): BTT (*n* = 27), high-risk PTC (*n* = 12), low/intermediate-risk PTC (*n* = 43), and PDTC/ATC (*n* = 7). *P*-values (***p*< 0.01; *** *p*< 0.001), Fisher’s exact test results comparing number of samples with and without REα and REβ expression in each tumor tissue type.


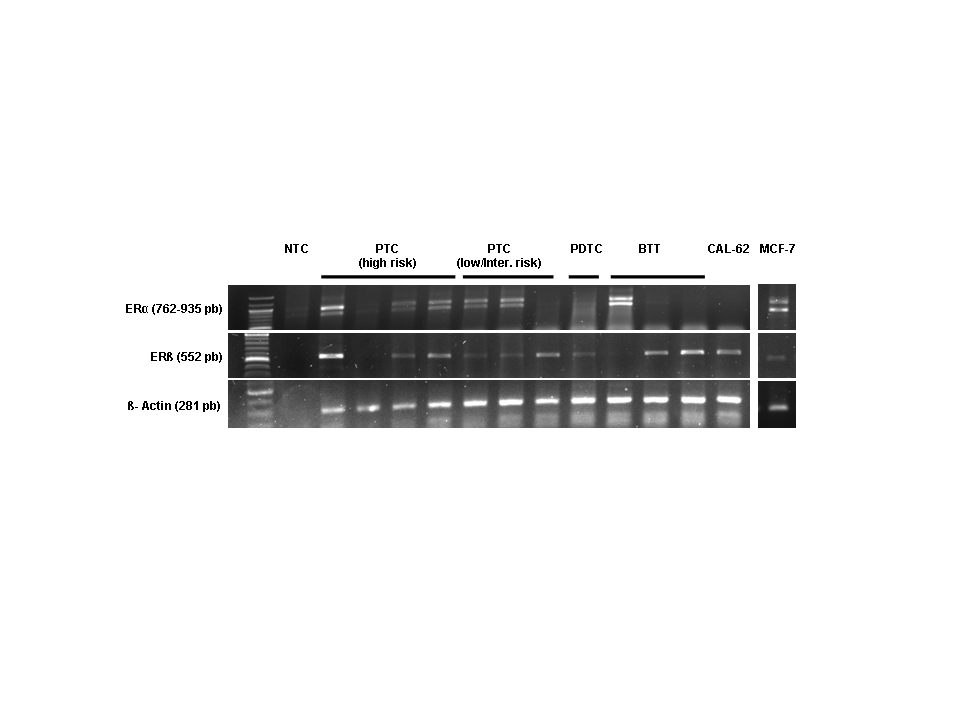


Representativeelectrophoresisagarose gel showing of the PCR amplicons results of the*oestrogen receptor*s isoforms (ERα and ERß)fromdifferenthumanthyroidtissuesamplesand MCF-7 cells. NTC (non template control)
